# Emerging Role of Adipocyte Dysfunction in Inducing Heart Failure Among Obese Patients With Prediabetes and Known Diabetes Mellitus

**DOI:** 10.3389/fcvm.2020.583175

**Published:** 2020-11-02

**Authors:** Alexander E. Berezin, Alexander A. Berezin, Michael Lichtenauer

**Affiliations:** ^1^Internal Medicine Department, State Medical University, Ministry of Health of Ukraine, Zaporozhye, Ukraine; ^2^Internal Medicine Department, Medical Academy of Post-Graduate Education, Ministry of Health of Ukraine, Zaporozhye, Ukraine; ^3^Division of Cardiology, Department of Internal Medicine II, Paracelsus Medical University Salzburg, Salzburg, Austria

**Keywords:** adipose tissue, cardiac and vascular remodeling, heart failure, co-morbidities, biomarkers

## Abstract

Adipose tissue dysfunction is a predictor for cardiovascular (CV) events and heart failure (HF) in patient population with obesity, metabolic syndrome, and known type 2 diabetes mellitus. Previous preclinical and clinical studies have yielded controversial findings regarding the role of accumulation of adipose tissue various types in CV risk and HF-related clinical outcomes in obese patients. There is evidence for direct impact of infiltration of epicardial adipocytes into the underlying myocardium to induce adverse cardiac remodeling and mediate HF development and atrial fibrillation. Additionally, perivascular adipocytes accumulation is responsible for release of proinflammatory adipocytokines (adiponectin, leptin, resistin), stimulation of oxidative stress, macrophage phenotype switching, and worsening vascular reparation, which all lead to microvascular inflammation, endothelial dysfunction, atherosclerosis acceleration, and finally to increase in CV mortality. However, systemic effects of white and brown adipose tissue can be different, and adipogenesis including browning of adipose tissue and deficiency of anti-inflammatory adipocytokines (visfatin, omentin, zinc-α2-glycoprotein, glypican-4) was frequently associated with adipose triglyceride lipase augmentation, altered glucose homeostasis, resistance to insulin of skeletal muscles, increased cardiomyocyte apoptosis, lowered survival, and weak function of progenitor endothelial cells, which could significantly influence on HF development, as well as end-organ fibrosis and multiple comorbidities. The exact underlying mechanisms for these effects are not fully understood, while they are essential to help develop improved treatment strategies. The aim of the review is to summarize the evidence showing that adipocyte dysfunction may induce the onset of HF and support advance of HF through different biological mechanisms involving inflammation, pericardial, and perivascular adipose tissue accumulation, adverse and electrical cardiac remodeling, and skeletal muscle dysfunction. The unbalancing effects of natriuretic peptides, neprilysin, and components of renin–angiotensin system, as exacerbating cause of altered adipocytokine signaling on myocardium and vasculature, in obesity patients at high risk of HF are disputed. The profile of proinflammatory and anti-inflammatory adipocytokines as promising biomarker for HF risk stratification is discussed in the review.

## Introduction

Abdominal obesity (AO) and diabetes mellitus (DM) remain global public health problems that are associated with a high risk of premature death and disabilities in the general population (1). The global AO and DM epidemic affects 2 billion people and 415 million people worldwide (1, 2). Prevalence of both conditions continue to increase worldwide, resulting in a higher burden of cardiovascular (CV) diseases due to acceleration of atherosclerosis, endothelial dysfunction, and microvascular inflammation (3, 4). Multiple observation studies have shown that AO and DM were associated with increased risk of heart failure (HF) manifestation regardless of other conventional CV risk factors (5–7). The results of the Framingham Heart Study have unveiled that the population-attributable risk of HF related to AO was 5% for men and 7% for women for each increment of 1 in body mass index (BMI) and to DM was 6% in men and 12% in women (7, 8). The NHANES (National Health and Nutrition Examination Survey) Epidemiologic Follow-Up Study has shown that DM independently predicted HF (9). Moreover, mild elevations in fasting glucose levels and insulin resistance (IR) abnormalities even in the absence of overt DM were associated with a dramatic increase in the risk of HF development (10). A meta-analysis of 77 prospective studies, which included patients with DM, AO, and HF, has shown that individuals with DM were at an increased risk of developing HF, and there was evidence of increased HF risk even within the prediabetic range of blood glucose among AO patients (11).

Despite that both AO and DM predominantly corresponded to HF with preserved (≥50%) ejection fraction (HFpEF), the prevalence of both conditions among patients having HF with reduced (<40%) ejection fraction (HFrEF) is quite high. The interrelation of mortality with AO and DM in patients having various phenotypes of HF remains to be debated. Although 90-day post-discharge cumulative all-cause mortality among HFrEF patients having DM was higher than those who had HFpEF, there were no significant differences in the overall cumulative all-cause mortalities in DM patients with various phenotypes of HF (12). However, among non-DM individuals with HFrEF, all-cause mortality was higher than those who had HFpEF or HF with midrange (40–49%) ejection fraction (12–14). Additionally, HF patients with known DM and microvascular complications had an increased risk of hospitalization, and prognostic significance of DM for complications including neuropathy, nephropathy, and retinopathy was higher in patients with HFpEF than those who had HFrEF (15).

In addition, previously, overweight and AO were found to be associated with substantially improved survival in HF patients when compared to normal-weight HF patients (16). This phenomenon has been termed the “obesity paradox,” and it was observed for all-cause mortality (16). Although the obesity paradox was also established in a wide range of patients having other CV diseases, including stable angina, atrial fibrillation, and hypertension; this phenomenon has been determined in retrospective studies in which AO was qualified according to BMI criteria, but not other indices of adiposity, such as dual-energy X-ray absorptiometry (17–19). Probably, AO in patients might present to clinicians prior to HF occurrence, leading to lag-time bias. Yet, AO patients may demonstrate significant variability in CV risk factor profile and have an attenuated neurohumoral activation [renin–angiotensin–aldosterone system (RAAS), natriuretic peptides], which could favor a better long-term prognosis. Later meta-analysis of 29 clinical trials has revealed that overweight was associated with lower CV mortality, but there were no significant differences in the cohorts of AO patients across other BMI (17). Nevertheless, there were no sufficient differences in mortality among AO patients having HFrHF and HFpEF (18, 19).

Despite controversial issues regarding an influence of AO on mortality among HF patients (16, 17), dramatic growth of AO prevalence is associated with soaring incidence of prediabetes and DM and consequently leads to increased CV risk among all people groups with different ages, in both sexes, in every racial and ethnic groups as well (18, 19). Abdominal adiposity corresponding to overexpression and secretion of adipocytokines, such as leptin, adiponectin, resistin, visfatin, omentin, zinc-α2-glycoprotein, and glypican-4, in white and brown adipose tissue, has been linked to DM and IR (20). Therefore, the systemic effects of white (WAT) and brown adipose tissue (BAT) can be different, and adipogenesis including browning of adipose tissue and deficiency of anti-inflammatory adipocytokines was strongly associated with adipose triglyceride lipase augmentation, altered glucose homeostasis, resistance to insulin of skeletal muscles, increased cardiac myocyte apoptosis, and lowered survival and function of progenitor endothelial cells, which could significantly influence on HF development, as well as end-organ fibrosis and multiple comorbidities (21). However, whether the adipocytokine dysfunction is crucial for adverse maladaptive cardiac and vascular remodeling and development of different phenotypes of HF is uncertain and is under investigation so far (22–24). Finally, the role of adipocyte dysfunction in the association between AO and survival advantage in HF is not certain. The aim of the narrative review is to summarize the evidence showing that adipocyte dysfunction may induce the onset of HF and support advance of HF through different biological mechanisms involving inflammation, epicardial and perivascular adipose tissue accumulation, adverse and electrical cardiac remodeling, and skeletal muscle dysfunction.

## Abdominal Obesity and Adipose Tissue Accumulation: Focus on HF Development

According to conventional views, adipose tissue accumulation is a result of disequilibrium between energy intake and energy expenditure. Adipose tissue comprises various depots including WAT, BAT, and other ectopic adipose tissues including thoracic, epicardial, abdominal, retroperitoneal, and perivascular adipose tissues. The phenotype of thoracic and epicardial adipose tissue resembles BAT, whereas both molecularity and functionality of abdominal, retroperitoneal, and perivascular adipose tissues do not distinguish WAT (24, 25).

**Figure 1:**
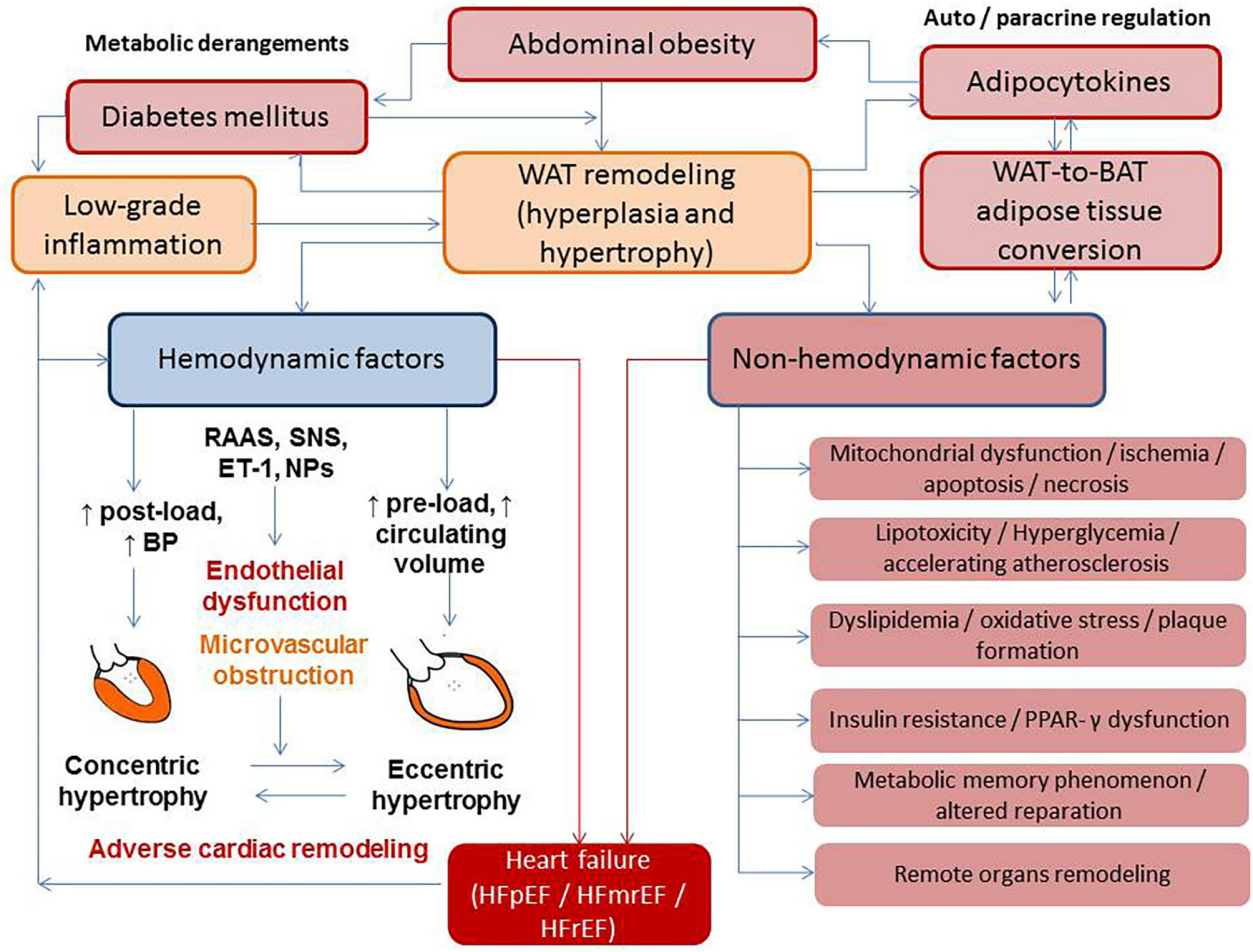
The interplay between metabolic derangements and heart failure development. BAT, brown adipose tissue; BP, blood pressure; ET, endothelin-1; HFpEF, heart failure with preserved ejection fraction; HFmrEF, heart failure with midrange ejection fraction; HFrEF, heart failure with reduced ejection fraction; NPs, natriuretic peptides; RAAS, renin–angiotensin–aldosterone system; SNS, sympathetic nervous system; PPAR-γ, peroxisome proliferator-activated receptor coactivator-1γ; WAT, white adipose tissue.

Development of AO corresponds to the conversion of fat-accumulating WAT into energy-dissipating functional BAT (25, 26). On the one hand, the interplays between the sympathetic nervous system (SNS), RAAS, endothelin and natriuretic peptide systems, and the thyroid–adrenal gland axis are strong contributors of adaptive hemodynamic responses that ensure the regulation of cardiac output, blood pressure, peripheral vascular resistance, fluid retention, water- and natriuresis, and consequently a balance between preload and after-load (Figure 1). On the other hand, these neurohumoral mechanisms mediate physiological BAT-related thermogenesis, energy expenditures, and WAT-to-BAT conversion in pathophysiological conditions, leading to transformation of metabolically non-active obesity to metabolically active obesity (27, 28). Moreover, adipose-derived angiotensin II contributes to circulating RAAS, kidney function, electrolyte and water homeostasis, and blood pressure regulation (28).

**Figure 2:**
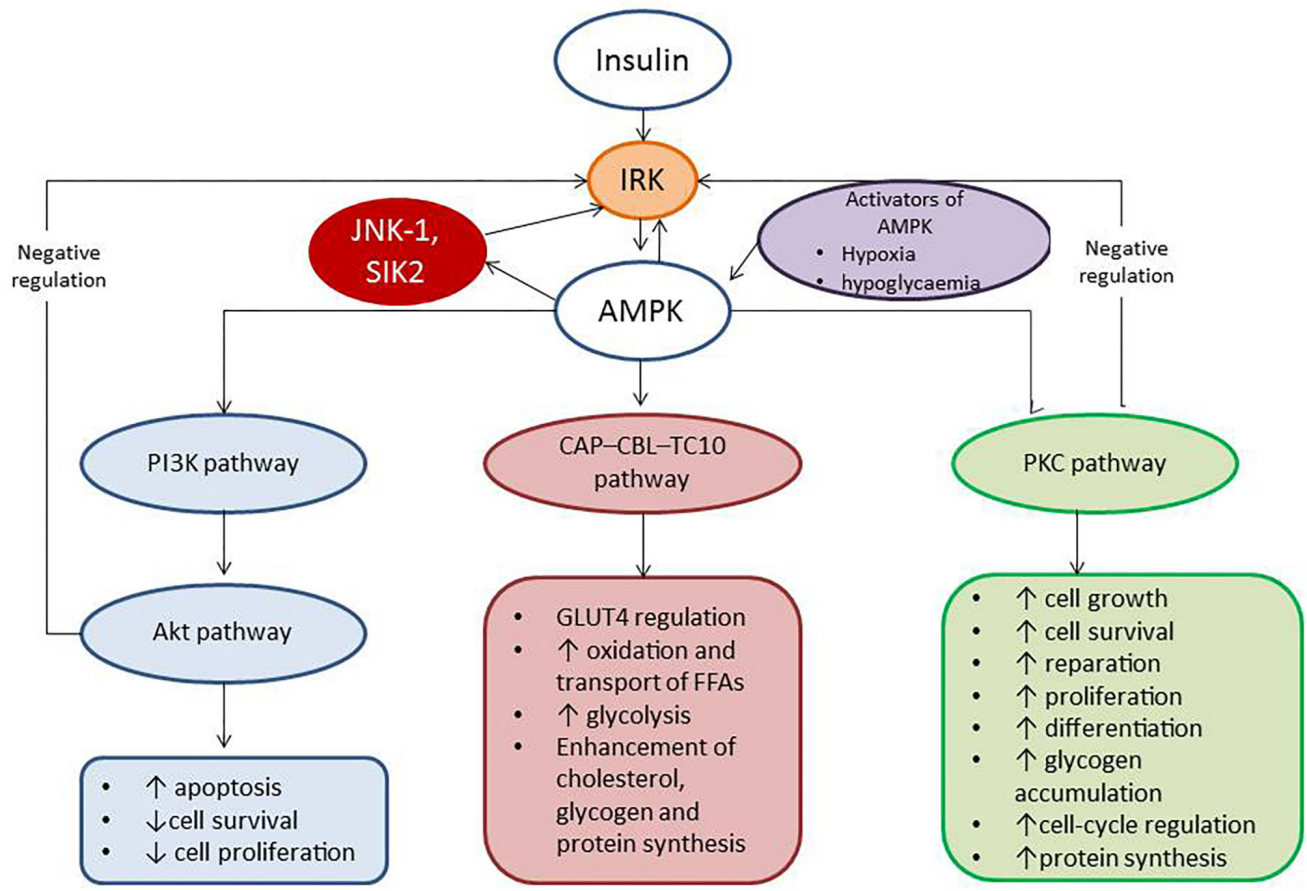
The role of insulin and AMPK in growth, differentiation, and survival of cells. AMPK, 5′-AMP–activated protein kinase; IRK, insulin receptor kinase; PI3K, phosphatidylinositol 3-kinase; CAP–CBL–TC10, the Cb1-associated protein (CAP)–casitas B-lineage lymphoma (CBL)–ras-like protein TC10; PKC, protein kinase C; JNK-1, c-Jun N-terminal kinase 1; SIK2, salt-inducible kinase 2.

In addition, IR is a result of the alteration of insulin and 5′-AMP–activated protein kinase (AMPK) signaling pathways, which regulate utilization of glucose and free fatty acids. Activation of AMPK leads to phosphorylation of phosphoinositide 3-kinases (PI3K) that recruits the Akt kinase, phosphoinositide-dependent kinase-1, and consequentlyThr308. The Akt phosphorylates several molecular targets including caspase 9, the proapoptotic B-cell leukemia/lymphoma 2, and ribosomal 70S subunit-S6 protein kinase, which regulate cell growth, differentiation, and survival (29). Finally, insulin acting through the insulin receptor kinase enables to negatively regulate signal transduction and triggers IR (29). Therefore, there are several mechanisms corresponding to AMPK ability to suppress cell growth, tissue differentiation, and reparation. In fact, AMPK indirectly declines the activity and production of several biosynthetic enzymes and directly blocks phosphorylation of p70S6K through targeting the tuberous sclerosis complex-2 and rapamycin complex 1 (TORC1) raptor. Consequently, the activation of AMPK enhances dual metabolic and cellular responses from target organs, such as attenuation cells metabolism and suppression of cell differentiation and growth (29). Figure 2 reports the role of insulin and AMPK in growth, differentiation, and survival of cells.

### White vs. Brown Adipose Tissue

WAT is defined as a heterogeneous tissue with high metabolic and regenerative plasticity. WAT is composed of subcutaneous and visceral adiposities and contains lipid-filled adipocytes and numerous non-adipocyte cell populations, which include mature and progenitor endothelial cells, uncharacterized stromal cells, adipocyte precursor cells, fibroblasts, and peripheral blood cells including several populations of antigen-presenting cells, such as T lymphocytes, mononuclear cells, and macrophages (30). Although metabolic (lipogenesis, lipolysis, fatty acid oxidation, amino acid, and sex steroid metabolism) and endocrine/paracrine (production of adipocytokines and natriuretic peptides) activities of WAT belong to adipocytes, non-adipocyte cell populations have demonstrated their pivotal roles in maintenance, growth, and function of WAT, as well as in metabolic and structural remodeling of remote organs (heart, skeletal muscles, liver, pancreas) and tissues (perivascular and pericardial adipose tissue) (31–34).

In response to appropriate stimuli, WAT can undergo a process of transformation into BAT. BAT is generally involved in adaptive sympathetically activated thermogenesis and energy homeostasis during cold exposure and after hyperphagia (35). Being metabolically active, BAT mediates thermogenesis through expression on their surfaces of uncoupling protein 1 (UCP1) having different phenotypes in classical brown adipocytes and beige/brite adipocytes (36). Yet, thermogenesis can be activated by certain stimuli including cold exposure, adrenergic compounds, or genetic alterations. Normally, there is inverse correlation between energy-dissipating activity of UCP1 in adult human beige/brite adipocytes and the BAT accumulation. Additionally, levels of UCP1 mRNA and other transcriptional regulators [peroxisome proliferator-activated receptor γ coactivator 1α (PGC-1α) and PR domain containing 16 (PRDM16)] in beige/brite adipocytes have been increased in parallel with PPAR-γ presentation (37). This finding suggests that the metabolic activity of BAT yields protective impact on body fat accumulation, glucose tolerance, and IR (37). Therefore, SNS, RAAS, and some adipocytokines (leptin, fetuin, visfatin) have also demonstrated powerful potency for activation and recruitment of beige/brite adipocytes and consequently maintenance of metabolic homeostasis and lipid metabolism (38–40). Promotion of BAT activity or the browning of WAT is associated with *in vivo* cold tolerance, increased energy expenditure, and protection against obesity and type 2 DM (41, 42). Overall, lower BAT activity that is frequently described as BAT dysfunction has been found as central player in regulation of metabolic homeostasis having a crucial role in the pathogenesis of AO, type 2 DM, and development of CV complications including adverse cardiac remodeling and HF (33, 42).

### Adiposity in HFrEF and HFpEF

Although AO is a risk factor of HF, there is differential interaction of AO with the occurrence of HFrEF and HFpEF (43). Indeed, the MESA (Multi-Ethnic Study of Atherosclerosis) study has reported the adiposity that was measured with anthropometrics (BMI and waist circumference) and an abdominal computer tomography were not associated with HFrEF, but VAT accumulation was strongly associated with HFpEF. Moreover, HFpEF patients had significantly more intramyocardial fat than HFrEF patients or non-HF controls. Interestingly, intramyocardial fat strongly correlated with left ventricular (LV) diastolic dysfunction parameters (predominantly echocardiographic E/e′ ratio) in HFpEF patients, but not in HFrEF patients, and this was independent of age, comorbidities, BMI, gender, and myocardial fibrosis (44). However, AO via myocardial steatosis, IR, and endothelial dysfunction influences cardiomyocyte hypertrophy and cardiac systolic and diastolic dysfunction (45). Indeed, insulin as an activator of PI3K /AKT alters titin-isoform composition and titin-based stiffness and could also contribute to altered cardiac diastolic function in patients with AO and DM (46).

In fact, AO is associated with a systemic proinflammatory state that induces oxidative stress and causes coronary microvascular inflammation, endothelial dysfunction, and altered cardiac and vasculature reparation. In addition, oxidative stress influences nitric oxide bioavailability, content of cyclic guanosine monophosphate and protein kinase G activity in adjacent cardiomyocytes (47). Nevertheless, low activity of protein kinase G mediates the development of cardiac hypertrophy and increases resting tension because of hypophosphorylation of titin and accumulation of collagen extracellular matrix (ECM) (47, 48). Moreover, there is evidence of the fact that hypophosphorylation of myofilament proteins and increased calcium sensitivity are the earliest molecular events in the development of HFpEF (48). Consequently, stiffness of myocardium and interstitial fibrosis ensures the development of diastolic filling abnormality and HF (49).

Thus, cardiac remodeling in HFpEF differs from HFrEF, in which remodeling is directly driven by primary loss of cardiac myocytes. The difference in the molecular mechanisms of the development of HF phenotypes corresponds well to the findings that explicitly explain a lack of rapid actin activation after inotropic stimulation in HFpEF because of hypophosphorylation of Ca^2+^-dependent thin filaments (50, 51).

## Adipocyte Macrophage Phenotypes and Altered WAT/BAT Activity

**Figure 3:**
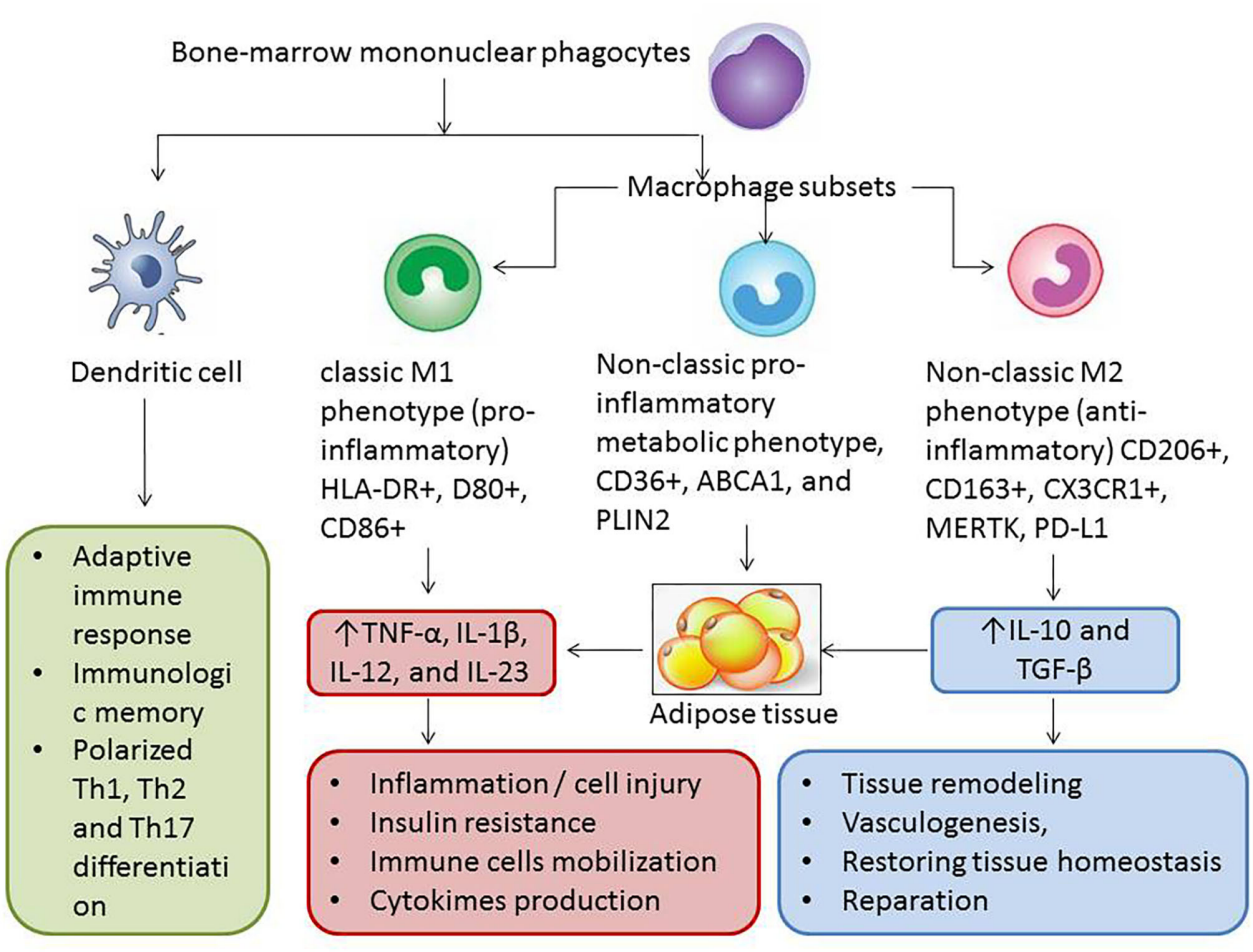
Interrelation between several phenotypes of macrophages, dendritic cells, and adipose tissue. The proportion of M2 macrophages is higher in BAT than in WAT and consequently lower mass of BAT in patients with AO, and type 2 DM is associated with weak anti-inflammatory ability of non–adipose tissue cells including M2 macrophages and regulatory T cells. As a result of the modification of immune phenotypes of adipose tissue macrophages and increase in WAT in patients with diabetes and patients with metabolically active obesity, the effect of M1 macrophages begins to prevail. It leads to microvascular inflammation, accelerating atherosclerosis, endothelial dysfunction, insulin resistance, skeletal muscle dysfunction, and finally cardiac and vascular remodeling and HF. TNF, tumor necrosis factor; IL, interleukin; TGF, transforming growth factor; ABCA1, ATP-binding cassette subfamily A member 1; PLIN2, perilipin-2; PD-L1, programmed cell death 1 ligand; CX3CL1, fractalkine.

Remarkable plastic properties of mature WAT/BAT adipocytes are supported by several stimuli, such as a lipolysis, liposecretion, low-grade inflammation, growth factors (transforming growth factor β, fibroblast growth factor), which mediate survival, migration, and (trans)differentiation of various stromal cells, such as preadipocytes, endothelial cells, fibroblasts, macrophages, and immune cells. There is evidence that the adult adipocytes of WAT turn to fibroblast-like elements (rainbow adipocytes) and mediate polarization of the macrophages with anti-inflammatory properties to classical M1 macrophages (52). Yet, macrophages with proinflammatory potencies are attracted to WAT in a result of adipose tissue–related cytokines [adiponectin, tumor necrosis factor α (TNF-α), interleukin 6 (IL-6)] and growth factors (transforming growth factor β) (53). M1 macrophages synthase and release a wide range of proinflammatory cytokines, such as TNF-α, IL-6, and monocyte chemoattractant protein 1 (MCP-1), and thus contribute to the development of microvascular inflammation, IR, skeletal muscle dysfunction, and accelerating atherosclerosis. Therefore, the inflammasome system being activated in stressed mature adipocytes of WAT regulates transdifferentiation of adipose tissue macrophages with non-classical phenotypes to classical macrophages and thereby stimulates apoptosis of mature adipocytes (53, 54). The debris of apoptotic adipocytes are reabsorbed by adipose tissue macrophages and induce a chronic low-grade inflammation, which potentially contributes to lower BAT activity, decreased mass of BAT in result of its transformation to WAT, development of IR, and development of type 2 DM (Figure 3). Unlike non-classical anti-inflammatory macrophages, adipose tissue dendritic cells support the adaptive immune reaction and proapoptotic activity through T_H_2/T_H_17 polarization. Additionally, M2 macrophages are able to induce regulatory T cells and produce IL-10 giving a tolerance to T_H_1 activation (55). Overall, the proportions of the classic and non-classic proinflammatory macrophages in adipose tissue among obese patients are higher than those of lean healthy individuals whose WAT contains increased number of macrophages with a less inflammatory M2 phenotype.

Interestingly, large amounts of macrophages are accumulated in WAT/BAT among obese and lean individuals by different mechanisms. WAT regulates adipose tissue macrophage differentiation through MCP-1/C-C chemokine receptor 2 pathways, but BAT mediates macrophage accumulation via TNF-α/mitogen-activated protein (MAP) kinase/nuclear factor κB (NF-κB) signaling, while the triggers for the process can be similar (56). In fact, increased local extracellular lipid concentrations are considered as a one of the important molecular triggers for WAT macrophage accumulation. The stromal adipose tissue cells are also involved in metabolic modification of the adipose tissue functions. For instance, the animal model of obese has revealed that an expansion of the ectopic adipose tissue was associated with an increase in serum ketone body concentration in circulation and reduced ratio of proinflammatory M1-like adipose tissue macrophages to anti-inflammatory M2-like macrophages (54, 55). Moreover, some key signal transductors that presented on the surfaces of macrophages influence variables depending on macrophage phenotypes. To note, G-protein–coupled receptor 43 (GPR43) transduce local TNF-α signaling derived from steady-state adipose tissue to macrophages. In fact, M2 macrophages, which were stimulated by TNF-α through GPR43-involving mechanism, supported WAT homeostasis and increase in metabolic activity, but M1 macrophages did not (56). Probably, functional heterogeneity of main and ectopic types of adipose tissues including perivascular and pericardial localization can relate to variability presentation of molecular receptor signal transductors (57, 58). However, adipose tissue dysfunction and elevated levels of TNF-α are coordinated by polarized macrophages (59, 60).

**Figure 4:**
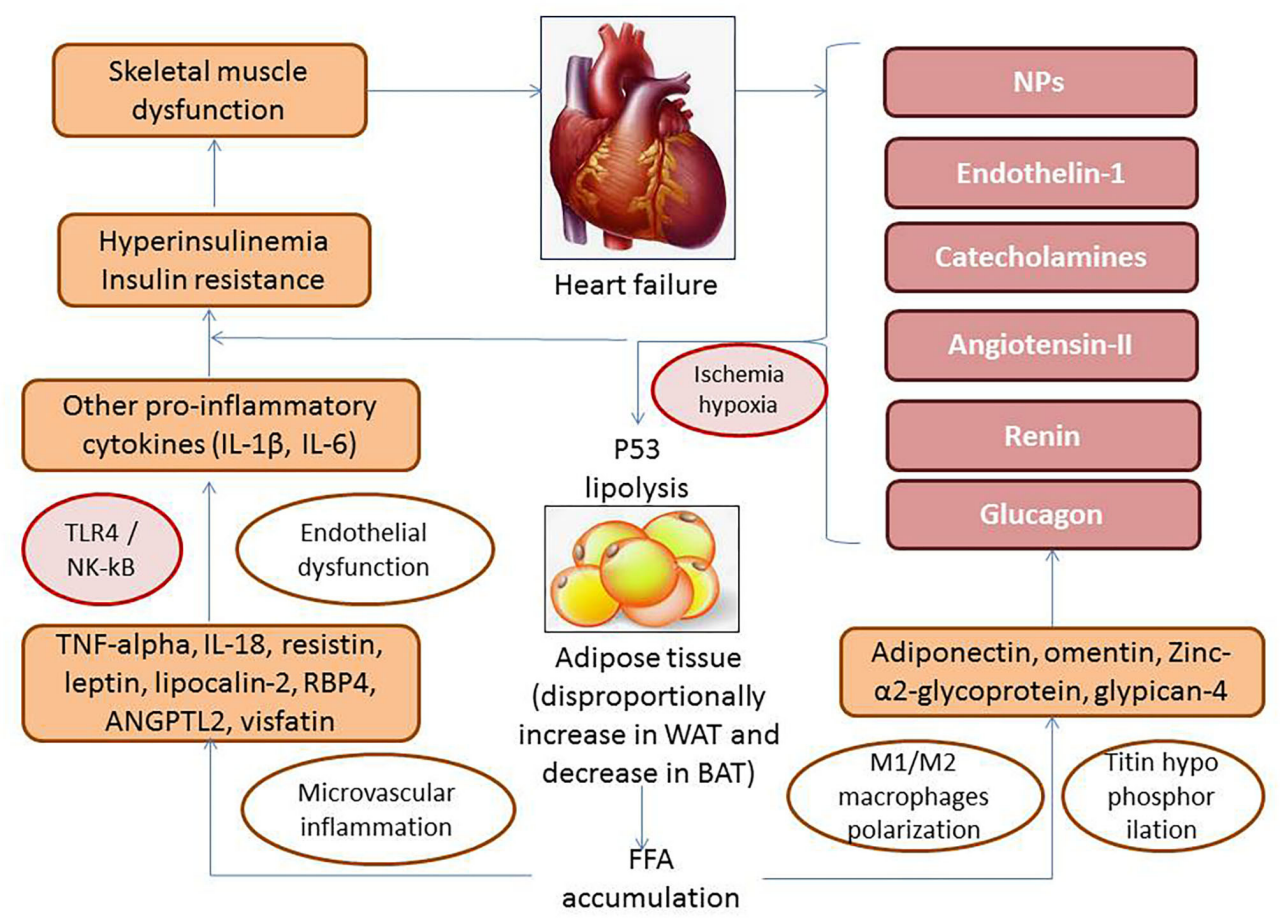
The role of free fatty acids–related stimulation of p53 and local production of adipocytokines in pathogenesis of HF. TLR4, toll-like receptor-4; NF-κB, nuclear factor κβ; TNF-α, tumor necrosis factor α; WAT, white adipose tissue; BAT, brown adipose tissue; IL, interleukin; RBP4, retinol-binding protein-4; ANGPTL2, angiopoietin-related protein 2.

The interplay between WAT adipocytes and macrophages carries out through autocrine/paracrine mechanisms including free fatty acids–related stimulation of p53 and local synthesis of TNF-α that establishes a vicious cycle aggravating inflammatory influences on target organs, such as the skeletal muscles, heart, liver, kidney, vasculature, and the adipose tissue (Figure 4). Indeed, free fatty acids accumulated in WAT in result of lipolysis induce GPR43, toll-like receptor-4 (TLR4) and activate c-Jun N-terminal kinase (JNK)–related proinflammatory pathways in antigen-presenting cells including macrophages, CD11c^+^ cells, which is associated with WAT inflammation (61). Mature adipocytes are destroyed by macrophages becoming the source of proinflammatory cytokines and triggers for adipocytokines release, reactive radicals synthesis, and oxidized phospholipid production (62). However, impaired macrophage autophagy was observed as a central player in macrophage polarization that down-regulates local inflammation in WAT (63).

Locally produced by M1 macrophages, adipocyte-specific caspase-1 and proinflammatory cytokines (IL-1β, IL-6) through NF-κB activation influence endothelial progenitor cells and fibroblast precursors and alter endogenous reparation of vasculature. Therefore, proinflammatory cytokines directly impair the metabolic status of the skeletal muscle inducing development of IR and consequently the skeletal muscle dysfunction (64, 65). Thus, the metabolic status of WAT adipocytes was found a crucial determinant of macrophage-related proinflammatory condition (66, 67). On the other hand, polarized macrophages strongly contribute to molecular biology and metabolic dysregulation of adipose tissue by impairing both its function and ability to transdifferentiation (68).

## Other Non–Adipose Tissue Cells and WAT Inflammation

Adipose tissue also contains non-adipocyte cells, such as endothelial cells and their precursors, epithelial cells, fibroblasts, profibroblasts, vascular smooth muscle cells (VSMCs), and immune and antigen-presenting cells, which mediates WAT inflammation, regeneration, and stroma transformation and exerts pleotropic effect on adipocytes. The stroma of WAT is mainly produced by fibroblasts, which synthesize and release several components of ECM including collagen and elastin fibers, as well as fibronectins, laminins, tenascin, and proteoglycans (69). The matrix structure provides mechanical support of WAT and ensures endocrine function of adipocytes through regulation matrix metalloproteinase activity and expansion of non–adipose tissue cells including antigen-presenting cells, effector T cells, IL-10–producing FoxP3^+^ T regulatory cells, natural killer cells, mononuclear cells/macrophages, and various progenitor resident cells with different origin (70–72). Indeed, effector T_H_1 cells including CD8^+^ cytotoxic T cells being under control of adipose-resident M1 macrophages produce interferon-γ (IFNγ) and stimulate synthesis and secretion of TNF-α, the Janus kinase (JAK) signal transducer and activator of transcription (STAT3) signaling pathway (73). Additionally, the number of WAT regulatory T cells directly inhibits WAT infiltration of T_H_1 cells and attenuates the reconstitution of M1 to M2 phenotypes of adipose tissue macrophages (74).

Overall, T cells play an important role in the initiation and perpetuation of inflammation in adipose tissue (75). Still, sometimes macrophages appear within the adipose tissue; T cells release proinflammatory cytokines that can be found locally within the adipose tissue, and these cells contribute to further inflammatory cell activation. Vice versa, an increased infiltration of T cells and macrophages into the adipose tissue also influences adipocyte functions by regulating the secretion of adipokines (76, 77). In this immunological response, T_H_1 and T_H_2 cells release cytokines, e.g., IFNγ and IL-4 that affect T-cell subset differentiation. Furthermore, T_H_17 cells are strong mediators in local tissue inflammation and secrete IL-17 and also further cytokines such as IL-21, IL-22, and IL-23 (78). T_H_17 cell activation induces long-lasting tissue inflammation (79) via cytokine release, though; the role of some of these cytokines still remains unclear in obesity, especially in its early stages when individuals are still young. Animal studies have shown that ingestion of a high-fat diet induced a short-term increase of IL-17– and T_H_17-associated cytokines. Over a longer period of time, high-fat diet resulted in a decrease of IL-17, IL-22, IFNγ, TNF-α, and IL-4 (80). Higher levels of IL-17 were also reported in obese adults (80).

In addition, PPAR-γ-driven lipolysis supporting free radical production by adipocytokines and acting via TLR signaling and netrin-1–dependent mechanism violates the repair ability of the residential endothelial progenitor cells and mesenchymal stem cells that leads to worsening vascular structure and function, microvascular inflammation, and finally antigen-presenting cell infiltration of WAT (64, 81–84). Collectively, adipose-resident immune cells promoting the proliferation and differentiation of other non–adipose tissue cells ensure remarkable excess and remodeling of ECM that lead to adipose tissue dysfunction and disproportional production of anti-inflammatory and proinflammatory adipocytokines (85). As a result of these processes, the WAT infiltration by inflammatory cells becomes a source of inflammatory cytokines and oxidative stress factors causing perivascular inflammation, cardiac and vascular remodeling, and endothelial dysfunction with impaired bioavailability of nitric oxide, contributing to atherosclerosis acceleration, plaque instability, target organ perfusion abnormality, and HF manifestation (86).

## Adipose Tissue Dysfunction and Heart Failure

Adipose tissue functions as a key endocrine organ by releasing multiple adipocytokines having proinflammatory or anti-inflammatory activities (Table 1). Dysregulation in synthesis or releasing of adipocytokines owing to WAT dysfunction can contribute to the pathogenesis of both obesity and HF (194). On the one hand, leakage of free fatty acids from the adipocytes due to lipolysis directly contributes to apoptosis of non–adipose tissue cells, microvascular inflammation, and altered adipose tissue perfusion leading to hypoxia/ischemia and necrosis and thereby shapes multiple proinflammatory signaling pathways in adipocytes, fibroblasts, and immune cells (195–197). On the other hand, hypoxia, which is developed either in the result of relative reduction in perfusion of the hypertrophic adipocytes and extended adipose tissue stroma or an increase in utilization of oxygen in AO, is established trigger for WAT inflammation. In fact, hypoxia is associated with overexpression of proinflammatory genes including hypoxia-inducing factor-1 gene, free radical production, oxidative stress, and lipotoxicity in adipose tissue and exerts altered adipocytokine secretion shaping vicious circle and promoting IR, skeletal muscle wasting, cardiac and vascular remodeling, endothelia dysfunction, and finally development of HF (198–201).

**Table 1:** Adipocytokines involved in the pathogenesis of obesity and HF.

**Adipocytokine**	**Primary source of synthesis in human**	**Main biological function**	**Changes in obesity/type 2 DM (T2DM)**	**Role in HF**	**References**
Adiponectin	Adipocytes	Regulation of energy homeostasis, glucose and lipid metabolism	↓ Circulating levels and ↑ adiponectin-1 receptors depending on presentation of conventional CV risk factors	↑ Levels are predictor of all-cause, CV, HF-related mortality, adverse cardiac remodeling, functional activity, skeletal muscle wasting, and metabolic disorders in HF	(87–98)
Leptin	Adipocytes	Body weight, feeding behavior, and energetic metabolism	↑ Levels among AO and T2DM patients	↓ Circulating levels predict HF severity, cardiac hypertrophy, pump dysfunction, cardiac and kidney fibrosis	(99–115)
Resistin	Macrophages	Sensitivity to insulin and FFA oxidation	↑ Levels are associated with IR, and oxidative stress and inflammation	↑ levels correlate with the severity of HF and predict HF outcomes	(20, 116–130)
Visfatin	Adipocytes, macrophages	Energy homeostasis, anti-inflammatory effect	↑ Levels in serum correlate with IR, BMI	↑ levels in HFpEF, ↓ levels in HFrEF	(131–142)
Omentin	Adipocytes, macrophages	Regulation of adipocyte differentiation, maturation, energy metabolism, immune response, inflammation, and IR	↑ Levels in serum, ↓ levels in vascular endothelium	Predictor of hospital readmission and mortality in HF patients	(138, 143, 144, 144–151)
Zinc-α2-glycoprotein	Adipocytes	Promoting lipid metabolism, glucose utilization, and insulin sensitivity	↑ Levels in serum, which correlates with omentin-1	Predictor of early diastolic filling abnormality and LV hypertrophy	(152–161)
Lipocalin 2	Adipocytes, macrophages	Regulation of inflammation and fibrosis	↑ Serum levels in connection with IR, hs-CRP	↑ TNF-α secretion from adipocytes, enhancing inflammation and IR	(162–166)
ANGPTL2	Adipocytes	Regulation of insulin sensitivity	↑ Serum levels and expression in WAT	Microvascular inflammation, accelerating atherosclerosis	(167, 168)
Secreted frizzled-related protein 5	Adipocytes	Regulation of growth, proliferation, ECM remodeling,	↓ Proinflammatory WNT signaling	Skeletal muscle waist and adverse cardiac remodeling	(169–171)
Glypican-4	Adipocytes	Regulation of insulin sensitivity	↑ Serum levels and expression in WAT	↑ Serum levels and ↑ expression in cardiac tissues, WAT, vasculature correlates with IR and BMI	(172–179)
Retinol-binding protein-4	Adipocytes, macrophages, hepatocytes	Regulation of insulin sensitivity	↑ Serum levels in connection with IR	Predictor of T2DM and IR in patients with established HF	(180–183)
TNF-α	VSMCs, adipocytes, APCs	↑ Local and systemic inflammation, ↓ insulin signaling	↑ Serum levels in connection with IR and systemic inflammation	Predictor of HF-related clinical outcomes	(184–193)
IL-6	Adipocytes, APCs, SVFCs, VSMCs, hepatocytes	↑ Inflammation, signal transductor of TNF-α	↑ Serum levels in connection with IR and systemic inflammation	Predictor of mortality	(191, 192)

*IR, insulin resistance; APCs, antigen-presenting cells; ANGPTL2, angiopoietin-like protein 2; SVFCs, stromal vascular fraction cells; VSMCs, vascular smooth muscle cells; FFA, free fatty acids; hs-CRP, high-sensitivity C-reactive protein; ECM, extracellular matrix, ↑, increase; ↓, decrease*.

To our knowledge, the secretome of adipocytes contains not only secretory adipocytokines, but also extracellular vesicles (ECVs), which transfer wide-spectrum regulatory molecules including coding and non-coding RNAs that play pivotal role in the intraorgan communication between adipose tissue and CV system (202). Although cell-free RNAs in human serum have been present in higher concentrations to ECVs, half biotypes of coding and non-coding RNA (micro-RNA, transfer RNA, small yRNA, circular RNA, and long non-coding RNA) are transferred with ECVs. There is evidence of the assumption that different phenotypes of HF are the result of altered cardiac and vascular reparation due to certain epigenetic responses, which are yielded by AO and DM (24). The genetic material and active molecules that are in ECVs are transductors of epigenetic signals and thereby regulate remodeling (203). In addition, adipocytes, cardiac myocytes, the vasculature, and immune cells in AO and DM are connected with each other through specific ECVs carrying nucleic acids, proteins, lipids, and cellular metabolites (204). Although the number of various subtypes of ECVs especially originated from endothelial cells correlated well with BMI and Homeostasis Model Assessment of Insulin Resistance (HOMA-IR) (205), there are a limiting number of non-coding RNAs, such as hsa-miR-423-5p, rno-miR-16, rno-miR-20b, rno-miR-93, rno-miR-106b, rno-miR-223, hsa-miR-660-3p, hsa-miR-665, hsa-miR-1285-3p, and hsa-miR-4491, which was strongly associated with the development of HFpEF (206). Some of the muscle-specific circulating miRNAs (hsa-miR-423-5p, rno-miR-16, rno-miR-20b) and others contribute to interstitial fibrosis (hsa-miR-665, hsa-miR-1285-3p, and hsa-miR-4491), IR (miR-141-3p), and inflammation (miR-4763-3p) (24). Based on the current knowledge of RNA regulatory networks, multiple ECV-derived non-coding RNAs definitely ensure cell-to-cell communications and mediate tissue response, i.e., cardiac and vascular remodeling, endothelial dysfunction, and browning adipose tissue. However, the practical benefit of these findings is not fully clear and requires deep investigation in the future.

### Circulating Adipocytokines

The most biological relevant adipocytokines pertaining to WAT dysfunction associated with clinical outcomes among HF patients are adiponectin, leptin, resistin, omentin, visfatin, angiopoietin-like protein 2, zinc-α2-glycoprotein, glypican-4, lipocalin 2, secreted frizzled-related protein 5, retinol-binding protein-4, TNF-α, IL-6, and IL-18.

### Adiponectin

Adiponectin is one of most abundant adipocytokines, which circulates in high concentration in peripheral blood (87). Adiponectin is normally secreted by adipocytes and exists in several isoforms (multimeric forms, monomeric forms containing full-length and globular subforms, and some oligomers) and promote insulin sensitivity of liver, skeletal muscles, and adipose tissues including the ectopic ones and also exert cardiac tissue–protective capacity (88). The biological role of adiponectin has now been widely elucidated, and it properly relates to increase in free fatty acid β-oxidation, activation of glucose transport, and inhibition of gluconeogenesis in target organs, such as liver, heart, WAT, and skeletal muscle, through activation of AMPK, p38 mitogen-activated protein kinase, and PPAR-α, and thereby attenuates IR (89). The biological response is ensured by presentation of specific receptors (AdipoR1 and AdipoR2). However, there is a wide range of adiponectin-related pleotropic potencies, such as antidiabetic, antiatherogenic, anti-inflammatory, antiproliferative, and anti-ischemic properties (90). It has been suggested that adiponectin potentiates the expression of the anti-inflammatory IL-10 and thereby suppresses the NF-κB signaling pathway, leading to down-regulation of TNF-α-related inflammatory responses (90). The most important functional antagonists of adiponectin are leptin and resistin, the biological impact of which on energy homeostasis and target tissue metabolism is completely opposite to adiponectin (207).

The circulating levels of adiponectin in obese patients and patients with diabetes are lower than those of healthy volunteers (208). In fact, higher total and high-molecular-weight adiponectin levels have been associated with a significantly lower risk of type 2 DM (T2DM) (207, 208). Previous clinical studies have shown that total and high-molecular-weight adiponectin oligomers were inversely associated with body mass index, fasting glucose and insulin levels, triglyceride concentrations, HOMA-IR, and visceral fat accumulation (209–211). Therefore, low circulating adiponectin levels (<12.4 mg/L) have shown a strong link with the presence of conventional CV risk factors, such as smoking and hypertension (212).

Interestingly, patients with LV hypertrophy with asymptomatic diastolic dysfunction and individuals with HFpEF had lower circulating levels of adiponectin compared with those who had HFrEF (122, 213, 214). Elevated levels of circulating adiponectin and also increased expression of adiponectin in skeletal muscles have been found in HFrEF patients, but high levels paradoxically corresponded to poor clinical outcomes, CV, and all-cause mortality (215). These findings were accompanied with low tissue expression of the main adiponectin receptor and genes that were involved in the down-regulation of lipids and glucose metabolism (91, 92). Because of the metabolic abnormalities (such as, IR), aerobic capacity, submaximal exercise performance, exercise muscle intolerance, and muscle strength among HF patients were strongly associated with circulating adiponectin levels, it has been hypothesized that adiponectin and its receptors could be key players in the development and advancement of HF myopathy (216). There was positive correlation between plasma levels of brain natriuretic peptide (BNP) and adiponectin in patients with established HFrEF and those who were at higher risk of HF (93, 217). Moreover, BNP was found to be the main driver of circulating adiponectin in AO patients with coronary artery disease (CAD) regardless of HF (94). Based on these results, it has been suggested that elevated levels of adiponectin among HFrEF patients are compensatory adaptive mechanism that allows overcoming the metabolic dysregulation and adiponectin resistance to prevent HF progression. Indeed, HF-related impairment of perfusion in target organs including skeletal muscles, liver, heart, vasculature, and kidney is associated with uncoupling G-protein that is incorporated into structure of adiponectin receptors AdipoR1 (skeletal muscles, heart, vasculature, and kidney) and AdipoR2 (liver). AdipoR1 is a powerful regulator of PPAR-γ coactivator-1-α and mitochondrial Ca^2+^-dependent ionic channels and AMPK/SIRT1-signal pathway (95). In contrast, AdipoR2 ensures a transduction of tissue-protective signals through the ubiquitin–proteasome pathway and insulin receptor tyrosine phosphorylation, and microRNA-150, which counteract the G-subunit of AdipoR2, may contribute to adiponectin resistance HF (96). Therefore, tissue expression of AdipoR2 in advanced HF was found to be significantly decreased (97).

The disruption of both receptors abrogates an ability of adiponectin to bind them and potentiate glucose and lipid metabolism that leads to switching off aerobic glucose metabolism to anaerobic way of glucose oxidation and also altered uptake of free fatty acids to skeletal muscles, lipid peroxidation, mitochondrial dysfunction, and finally the development of IR (98). Although adiponectin can stimulate mitochondrial biogenesis and increase the oxidative capacity in skeletal muscles, oxidative, and mitochondrial stress diminishes the capability of adiponectin to improve glucose utilization and potentiate fatty acid β-oxidation (218, 219). Thus, adiponectin resistance appears to be a cause of both cardiac contractility dysfunction and skeletal muscle weakness (220). Although concise molecular mechanisms, by which resistance to adiponectin pertains to cardiac and skeletal muscle dysfunction, remain not fully elucidated, adiponectin levels in peripheral blood are promising biomarker for metabolic abnormalities in HFrEF patients strongly relating to clinical outcomes and survival.

### Leptin

Leptin is a multifunctional adipocyte-derived hormone, the receptors for which are widely expressed in numerous peripheral tissues and the hypothalamus, but not only in adipose tissue (221). The main biological role of leptin is regulation of body weight, feeding behavior, and energetic metabolism (99). Leptin acts as physiological antagonist of adiponectin through binding with appropriate receptors and activates JAK-STAT3 signal transduction pathway. Circulating leptin levels are normally higher in female to male, while there is a strong positive correlation between leptin concentration and adipose tissue mass, and obese individuals usually demonstrate higher levels of leptin to healthy volunteers (99). However, the development of AO and metabolic syndrome corresponds to hyperleptinemia and tissue leptin tolerance (100). There is evidence that increased hypothalamus levels of leptin facilitate a cognition and synaptic plasticity, but leptin resistance, in contrast, increases the risk of depression in patients with AO and T2DM (100). Administration of leptin was associated with the improvement of peripheral tissue sensitivity to insulin and the attenuation of energy homeostasis (222, 223).

As a cytokine with structural resemblance to IL-2 and growth hormone 1, leptin modulates both innate and adaptive immune responses and proinflammatory capacity of T_H_1 lymphocytes and macrophages acting as a stimulator of the JAK2–STAT3 pathway and thereby increases the production of several proinflammatory cytokines, such as IL-2, IFNγ, TNF-α, and CC-chemokine ligands (CCL3, CCL4, and CCL5) (101, 224, 225). Therefore, leptin significantly increases migratory and proliferative ability of mononuclear cells and monocytes and also induces the secretion of free radicals enhancing oxidative stress (102).

Although there is a wide range of evidence of participation of leptin in direct and indirect regulation of cardiac function, the exact understanding whether leptin influences detrimentally and in contrary positively on myocardium is not clear (103). On the one hand, leptin in animal models demonstrated proinflammatory activity, which was associated with remodeling of ECM, WAT inflammation, endothelial dysfunction (104, 105). On the other hand, there is evidence that leptin was able to inhibit apoptosis of cardiac myocytes and reduce severity of myocardial dysfunction in acute myocardial infarction model and ensured antiproliferative effects through stimulation of cardiac STAT3, PI3K, and Akt activity and mitochondrial function, and also leptin stimulated vascular reparation via nitric oxide–p38 MAP kinase–dependent mechanism (106–108). Additionally, non-canonical leptin signaling pathway has been found by which leptin interferes with epidermal growth factor receptors and thereby ensures antiproliferative response (109).

The patients with known HF have yielded increased circulating levels of leptin, depending on sodium retention and plasma volume expansion, whereas abundant results of the leptin serum level measurements are conflicting (110, 111). It has been hypothesized that the synthesis of leptin in HF pertains to cardiac and renal fibrosis and WAT and microvascular inflammation and that leptin-mediated neurohormonal and proinflammatory activation may enhance the expression of SGLT2 in the kidney tubules. Thus, SGLT2 inhibitors exert tissue protection by diminishing leptin-related inflammation and suppression of leptin synthesis in WAT, but not only by natriuretic actions (112). The next explanation affecting the role of leptin in the pathogenesis of HF pertains to the deleterious interaction of leptin, aldosterone, and neprilysin in HF patients with AO or T2DM (113). Perhaps, SNS and neprilysin overactivity among obese patients enhances the production of leptin and other proinflammatory adipokines and accompanies with altered natriuretic peptide clearance and adiponectin synthesis that contributes to HF progression (114). Additionally, there is hypothesis that leptin as a prohypertrophic factor exerts cardiac-protective effect, and its release from adipocytes is a maladaptive response against HF-related inflammatory activation (115). However, leptin representing a link between AO, T2DM, and HF, is a CV risk biomarker requiring more precise understanding of matter of these relationships.

### Resistin

Resistin is a low-molecular-weight adipocytokine contributing to IR, inflammation, and oxidative stress (116). The main biological effects of resistin have been executed through various molecular targets (free fatty acid transport protein 1, acetyl-CoA carboxylase, and AMPK, CD36) and affected the attenuation of glucose metabolism, inhibition of free fatty acid β-oxidation, and uptake (20). In humans, resistin is predominantly expressed and secreted by macrophages due to stimulation by proinflammatory cytokines (117). Therefore, resistin was found to promote microvascular inflammation, endothelial dysfunction, VSMC proliferation, and plaque formation (118). Although serum resistin levels did not demonstrate a link with a risk of non-fatal myocardial infarction in CAD patients and did not reduce infarct size, the highest quartile of resistin concentrations were found as an independent predictor of an increased risk of HF development (119, 120). The MESA study has revealed that incidences of CV disease, CAD, and HF showed strong, independent association with resistin levels in general population (121).

Previous studies have revealed that increased serum levels of resistin were associated with the IR, T2DM, AO, and CV diseases (226, 227), while there were no significant correlations between resistin levels and echocardiographic parameters including LVEF, Gensini score index, angiographic parameters, and severity of atherosclerosis (228). Both the Framingham Offspring Study and the Health ABC Study have shown that the serum levels of resistin independently corresponded to a high risk of adverse CV outcomes and worsening kidney function among the patients with HF, but adiponectin levels did not (122, 123). Moreover, the reduction of kidney function was the main cause of the elevation of circulating resistin level than declining cardiac pump function (124). There are controversial results of clinical studies pertaining to an association of resistin levels and HF-related outcomes and all-cause and CV mortality. In patients with non-ischemic dilated and inflammatory cardiomyopathy, resistin independently predicted the incidence of HF (125). In addition, resistin had a predictive ability for HFpEF, but not for HFrEF, in terms of morbidity and mortality (126). In the Bio-SHiFT study, an interrelation between serum resistin levels and HFrEF clinical outcomes during 2.2 years of follow-up was not found (127). In contrast, serum resistin levels have exhibited strong correlation with serum markers of ECMs (type III amino terminal propeptide of procollagen, matrix metalloproteinase-2, tissue inhibitor of metalloproteinase 1), BNP, apelin, and mortality in HFrEF patients (128). Thus, resistin rather explains an interrelation between metabolic comorbidities, inflammation, and HF than independent impact on a nature evolution of HF (129, 130).

### Visfatin

Visfatin is an anti-inflammatory adipokine enzyme (also known as nicotinamide phosphoribosyltransferase and pre–B cell colony-enhancing factor) having growth factor activity, which is involved in the biosynthesis pathway of NAD^+^ (131). Visfatin is actively secreted by macrophages and adipocytes and is found in the circulation and in ECMs in which it regulates the oxidative stress, immune response, apoptosis, and inflammation through Sirt-1–dependent and MAP kinase ERK1/2-related pathways (132, 133). The translocation of NF-κB and suppression of NF-κB visfatin significantly reduced the production of matrix metalloproteinase-8 and thereby diminished remodeling of ECM (134). In physiological condition, visfatin regulates thermogenesis in BAT via increase in UCP-1 levels in BAT adipocytes (229). Therefore, visfatin binds to the insulin receptor-1 and exerts an insulin-like effect (230). Being adipocytokines with various pleiotropic effects visfatin exerts IR, inhibits WAT oxidative stress and inflammation, promotes vascular reparation, and suppresses ischemia-induced apoptosis of cardiac myocytes mainly through up-regulation of proinflammatory cytokines, such as TNF-α and MCP-1 (231). On the other hand, visfatin also up-regulates NF-κB in endothelial progenitor cells inducing apoptosis of these precursors leading to a decrease in the number of circulating endothelial progenitor cells with angiopoietic activity (232, 233).

The serum levels of visfatin increase significantly in patients with AO, T2DM, metabolic syndrome, acute myocardial infarction, and HFpEF and decrease in patients with HFrEF (135, 234, 235). Among patients with acute ST-elevation myocardial infarction, elevated serum levels of visfatin predicted composite major adverse CV events (136). There is evidence that increased serum levels of visfatin predicted restenosis after implantation of drug-eluting stent (137). Additionally, there is a positive correlation between plasma visfatin level with triglycerides and inverse correlation with high-density lipoprotein cholesterol level and omentin-1 in CAD patients with HFpEF (138, 139).

It has been suggested that production of visfatin in patients with HF is adaptive response, which is directly against the impairment of mitochondrial ultrastructure, activation oxidative stress and free radical production, and cell death in the myocardium (140, 141). However, serum visfatin concentrations in HFrEF patients corresponded to New York Heart Association classes, and they are significantly lower compared with healthy volunteers regardless of age, anthropometric features, and metabolic parameters (142, 149). Overall, whether visfatin exerts potential beneficial effects on myocardium, vasculature, and adipose tissue in HF is not fully understood and requires to be elucidated in the large clinical studies in the future.

### Omentin

Omentin is a 34-kD protein that is released from omental adipose tissue and involved in the regulation of adipocyte differentiation, maturation, energy metabolism, immune response, inflammation, and insulin sensitivity (143). There are two homologous isoforms (omentin-1 and omentin-2) of omentin in circulation, and omentin-1 is the main isoform (144). Omentin-1 acts through AMPK/Akt/NK-κB/MAP kinase (ERK, JNK, and p38) signaling systems mediating anti-inflammatory, antioxidative, and angiopoietic effects (144). It has been found that omentin-1 exerts cardiac-protective effect apart from direct cardiac myocytes protection mediating cross-talk between WAT and myocardium (145). Omentin-1 exhibited sufficient activity against an oxidation of low-density lipoproteins and prevented foam cell occurrence via down-regulation of CD36, scavenger receptor class A, and acyl-CoA-cholesterol acyltransferase-1 and up-regulation of neutral cholesterol ester hydrolase in activated macrophages (146). Also, omentin-1 decreased angiotensin II–induced migration of monocytes/macrophages and platelet-derived growth factor BB–induced proliferation of VSMCs (138). To note, omentin-1 levels were markedly low expressed in coronary artery endothelium and epicardial adipose tissue, while circulating levels of omentin-1 and its expression in plaques were increased (147).

Probably, lowered levels of omentin-1 and increased concentrations of visfatin may implicate in the occurrence of CAD in AO patients (147, 148). The population-based EPIC-Potsdam study has shown that serum levels of omentin-1 were not significantly related to HF risk, but they were associated with a risk of CAD in the general population (147). Interestingly, patients with HFrEF demonstrated higher levels of omentin-1 compared to those who had HFpEF (148). Therefore, the elevated circulating levels of omentin-1 were mildly and positively associated with cardiac volumes and systolic function and negatively correlated with adiponectin, high-sensitivity C-reactive protein, and N-terminal pro-BNP (NT-proBNP) in HF patients (148, 149). In addition, the elevated levels of omentin-1 were an independent predictor of weight gain in patients with acutely decompensated and chronic HF who had less mortality rate and hospital readmission regardless of leptin and NT-proBNP levels (150, 151). However, the primary cause of a positive impact of omentin-1 on mortality among HF patients remains uncertain.

### Zinc-α-2-Glycoprotein

Zinc-α-2-glycoprotein is an adipocytokine, which belongs to the class I MHC protein and is released by epithelial cells and adipocytes (152). Zinc-α-2-glycoprotein binds with the dansylated C11 fatty acid 11-(dansylamino)undecanoic acid on the surface of target cells (adipocytes, skeletal muscles) and regulates lipid metabolism and sensitivity to insulin (153). The pleiotropic effects of zinc-α-2-glycoprotein relate to negative regulation of fibrosis and inflammation through the suppression of the synthesis of several proinflammatory cytokines, such as S100A1 (154, 155).

Zinc-α-2-glycoprotein is involved the development of AO and T2DM. Previous studies have shown that zinc-α-2-glycoprotein was a better predictor for IR than HOMA-IR index (156–158). Zinc-α-2-glycoprotein acts via interaction with p-ERK and TGF-β1, promoting proliferation of endothelial precursors, suppression of low-grade inflammation, regulation of metabolism of ketone bodies, and increased expression of visfatin in target cells (159, 160). There is evidence that zinc-α-2-glycoprotein prevents cardiac hypertrophy and improves diastolic performances probably due to attenuation of cardiac fibrosis (161). The cardioprotective ability of the cytokine requires to be clearly elucidated.

### Lipocalin 2

Lipocalin 2 (neutrophil gelatinase-associated lipocalin) is released by various cell types and belongs to the lipocalin protein superfamily (162). It is widely expressed in adipose tissue and is responsible for inflammation and fibrosis. There is evidence that overexpression of lipocalin-2 in WAT is under control of up-regulated IL-1β (163). Circulating levels of lipocalin 2 positively correlated to adiposity, hyperglycemia, IR, ECM remodeling, matrix metalloproteinase activity, and high-sensitivity C-reactive protein (164–166). Although lipocalin-2 exerted inflammatory, proliferative, and fibrotic response in myocardium and kidney (236), the role of this biomarker in adverse cardiac remodeling and HF occurrence is not clear. It has been reported that lipocalin-2 supported priming and activation of NLRP3-inflammasome and releasing HMGB1 from cells, leading to increase in circulating levels of IL-1β, IL-18, and caspase-1 activation (167, 237). Finally, microvascular inflammation and cardiac fibrosis are the most expected causes that contribute to the deteriorating impact of lipocalin-2 on myocardial structure and kidney function.

### Angiopoietin-Like Protein 2

Angiopoietin-like protein 2 is a multifunctional proinflammatory adipocytokine that promotes IR and widely expressed in the WAT (238). Increased circulating levels and overexpression of angiopoietin-like protein 2 were found in patients with AO and T2DM (168). The ability of this molecule to modulate vascular permeability and induce microvascular inflammation with respect to HF development is investigated.

### Secreted Frizzled-Related Protein 5

Secreted frizzled-related protein-5 is a novel adipocytokine, expressed in cardiomyocytes, fibroblasts, and adipocytes (169). It suppresses Wnt/β-catenin signaling and is involved in embryonic development, proliferation, vascular permeability, atherosclerosis, and apoptosis (170). Secreted frizzled-related protein 5 is down-regulated in HF patients and plays a pivotal role in HF-induced skeletal muscle dysfunction, cardiac fibrosis, and ECM remodeling through interaction with TGF- β1 (170, 171). This biomarker appears to be promised for further investigation pertaining to adverse cardiac remodeling and prognostication of HF development.

### Glypican 4

Glypican 4 is novel adipocytokine that belongs to the heparan sulfate proteoglycan family and is released by adipocytes. It plays an important role in the regulation of glucose tolerance and enhancement of insulin signaling (172). Glypican 4 in myogenic regulatory factor is responsible for skeletal muscle hyperplasia and hypertrophy, as well as cardiac remodeling and myocardial hypertrophy (173). Additionally, glypican 4 regulates Rac activation to maintain polarized actin-rich lamellipodia in ECMs, and it is crucial for efficient migration of endodermal cells into ECMs (174).

The serum levels of glypican 4 are progressively increased in patients with AO, metabolic syndrome, and T2DM in connection with increasing body mass index, waist circumference, waist-to-hip ratio, and total WAT mass (175–177). It has been proposed to measure the serum levels of glypican 4 to predict CV risk (178). In HF patients, serum levels of glypican 4 predicted the endurance training, and thereby it could be a novel target for biomarker-based therapy of HF (179).

### Retinol-Binding Protein 4

Retinol-binding protein-4 is an adipose tissue–derived protein with prodiabetogenic effects, which is secreted by adipocytes and hepatocytes (180). Controversial data exist regarding the interrelations between serum levels of retinol-binding protein-4, IR, AO, T2DM, and CV complications including HF. For instance, Ulgen et al. (181) did not find the association of retinol-binding protein-4 levels with IR and other components of the metabolic syndrome. In contrast, Lee et al. (182) reported that there were significant associations between fasting glucose levels, insulin levels, HOMA-IR, and retinol-binding protein-4 concentrations in AO patients. It has been suggested that retinol-binding protein-4 can be involved in WAT/BAT distribution in obese patients, while this assumption requires to be widely investigated.

Serum levels of retinol-binding protein-4 were significantly higher in HF patients in comparison with healthy volunteers (183). Whether retinol-binding protein-4 is a predictor for HF nature evolution or HF-related risks is not fully understood.

### Tumor Necrosis Factor-α

TNF-α is well-known adipocytokine that is produced by VSMCs, adipocytes, and antigen-presenting cells and is involved in the regulation of local and systemic inflammation, immune response, and IR (239). TNF-α is not expressed in the normal myocardium, but it can be produced by cardiac myocytes or macrophages in response to volume overload, or it can be transported in cardiac tissue from remote sites of synthesis, such as WAT (184). Being a central inflammatory mediator TNF-α directly provokes cardiac remodeling and leads to cardiac dysfunction in patients with AO and T2DM and indirectly by induction of NO synthase (185). However, there is no well-documented evidence for the credible role of anti–TNF-α therapy in prevention of HF among AO patients with established rheumatoid arthritis (186). Indeed, some large international, randomized, placebo-controlled clinical trials (RECOVER [Research into Etanercept Cytokine Antagonism in Ventricular Dysfunction] and RENAISSANCE [Randomized Etanercept North American Strategy to Study Antagonism of Cytokines]) failed to reveal a powerful positive effect of anti-TNF therapy vs. placebo on clinical outcomes in HF patients (187). Now the anti-TNF therapy is not recommended to all HF patients (188).

On the other hand, muscle wasting, and cardiac cachexia contribute to HF progression, and simultaneously, they are driven by systemic inflammation supported by TNF-α (189). Previous clinical studies have shown that circulating levels of TNF-α and its secondary mediators IL-6 and IL-18 were significantly higher in HFrEF patients when compared with HFpEF and healthy subjects. Therefore, TNF-α, IL-6, and IL-18 have yielded significantly higher concentrations in ischemia-induced HF to HF that was associated with valvular heart disease and hypertension (190). Although there is strong association between mortality rate and serum levels of TNF-α and IL-6 among HFrEF patients, there is still no clear understanding how these cytokines contribute to HF and mediate their cross-talk with sympathetic system and WAT dysfunction (191–193). Finally, TNF-α is probably one of the promising biomarkers to predict skeletal muscle weakness during personifying HF therapy.

### Interplay Between the Adipocyte Dysfunction, Abdominal Obesity, and Survival Advantage in HF

Recent clinical findings have shown that dysregulation of adipocytokine production is a crucial factor contributing to the manifestation and progression of AO-induced metabolic and CV complications including HF (179, 181, 185, 193, 240). The impact of altered adipocytokine profile on CV remodeling in patients with several phenotypes of HF is remarkably variable and does not always relate to inducing inflammatory activation. The mechanisms that connect AO and HFpEF vary from obesity-induced hemodynamic changes to important biohumoral systems such as adipocytokines, RAAS and SNS, natriuretic peptide, and oxidative stress. Perhaps, altered adipocytokine profile may predict the occurrence of HFpEF and HFrEF, although the causative relation of AO to a risk of clinical outcomes in HFrEF requires to be deep elucidated. However, there is still no agreement regarding survival advantage in AO patients having HFrEF or HFpEF based on adipocytokine dysfunction (241, 242).

## Conclusion

HF is common complication of AO and T2DM often occurring as a result of adipocyte dysfunction and adipose tissue expansion. Adipocytokines is implicated in the sophisticated cascade of potentially reversible HF metabolic derangements, which can be effectively treated and probably accurately predicted by circulating biomarkers. The balance between the proinflammatory and anti-inflammatory cytokines that are involved in the metabolic regulation of WAT/BAT is an essential core element in understanding of a pivotal role of adipocytokine dysfunction in HF manifestation among obese patients and patients with diabetes. Clear molecular and functional mechanisms of adipose tissue dysfunction require to be elucidated in large clinical studies to open new perspectives in prediction of HF occurrence and development among patients with AO and T2DM.

## Author Contributions

All authors listed have made a substantial, direct and intellectual contribution to the work, and approved it for publication.

## Conflict of Interest

The authors declare that the research was conducted in the absence of any commercial or financial relationships that could be construed as a potential conflict of interest.

## Abbreviations

AMP: adenosine-monophosphate
AO: abdominal obesity
BAT: brown adipose tissue
BNP: brain natriuretic peptide
DM: diabetes mellitus
ECM: extracellular matrix
HF: heart failure
HFmrEF: heart failure with mid-range ejection fraction
HFpEF: heart failure with preserved ejection fraction
HFrEF: heart failure with reduced ejection fraction
HIF-1: hypoxia-inducing factor-1
JAK: Janus kinase
LV: left ventricular
NPs: natriuretic peptides
NK-κB: nuclear factor, κB
NT-proBNP: N-terminal pro-brain natriuretic peptide
RAAS: renin–angiotensin–aldosterone system
SNS: sympathetic nervous system
STAT: signal transducer and activator of transcription
STEMI: ST-elevation myocardial infarction
T2DM: type 2 diabetes mellitus
TGF- β1: transforming growth factor
UCP-1: uncoupling protein-1
WAT: white adipose tissue.
